# The implantable loop recorder: A tool that is "here to stay"

**Published:** 2002-01-01

**Authors:** Carel C Cock

**Affiliations:** Department of Cardiology, VU University Medical Center, Amsterdam, The Neteherlands

## Introduction

Syncope is a common clinical disorder accounting for 3 % for emergency room presentations and 1 % of hospital admissions [1]. Moreover it has not always a benign course, with mortality rates up to 33 % at 1 year in patients with a structural cardiac disorders [2-5]. In addition costs for investigation for syncope is substantial and about 25 % of all patients remain undiagnosed [6].

Investigation of patients with recurrent unexplained syncope may include electrocardiography and treadmill exercise testing, neurologic testing, tilt table testing, ambulatory Holter monitoring and electrophysiological testing.

This review will briefly discuss these current available strategies and focus on the usefulness of a recently introduced tool using an implantable loop recorder (ILR) in the treatment of patients with a recurrent unexplained syncope.

## Clinical history and physical examination

A careful history taking and clinical examination in addition to electrocardiography is essential for patients referred for recurrent syncope. History should focus on postural symptoms, palpitations, family history (long QT syndrome, Burgada syndrome, cardiomyopathy) and should include the use of medication particularly in the elderly patients [7-9]. Physical examination should focus on (orthostatic) blood pressure, cardiac murmurs and specific cardiac disorders e.g. aortic or mitral stenosis.The yield of these initial evaluation however is low with less than 50 % of patients having a primary diagnosis made.

## Electrocardiography

Electrocardiography is essential in the work-up of patients with unexplained syncope but may reveal a direct cause in only 5 % of patients. Pre-excitation patterns, a long QT- interval, the recently reported Burgada syndrome and characteristic features in patients with arrhythmogenic right ventricular dysplasia should all be considered [7-9].

## Echocardiography and exercise testing

Echocardiography may reveal structural heart disease e.g. cardiomyopathy but the diagnostic yield is low in patients without a cardiovascular history and normal physical examination. Although more than half of patients with unexplained syncope presented with an abnormal echocardiogram, no additional value could be demonstrated in a large series of patients reported by Recchia et al [10]. In addition a low diagnostic yield of exercise testing was found.

## Tilt table testing

Recently a large number of studies reported on the use of tilt table testing for patients with recurrent syncope [11-14] resulting in an expert consensus on the use of tilt table testing by the American College of Cardiology in 1996 [15]. An international classification for tilt testing for patients with induced neurocardiogenic syncope proposed in 1992 indicated that several subtypes (i.e. type 2a and type 2b) are candidates for pacing therapy for recurrent syncope. Head-up tilt table testing provides the highest yield from all diagnostic procedures in patients with recurrent syncope. However still the majority of patients cannot be detected using this test [1]. Connolly et al [16] recently studied the value of pacing in patients with neurocardiogenic syncope and reported a 85 % reduction of symptoms in patients randomly assigned to DDD pacing with a rate drop response algorithm. However Benditt [17] commented in an editorial referring to this study stating that "the relationship between observations during tilt table induced faints and the pathofysiology of spontaneous syncopal events in the same patients had yet to be studied adequately". Nonetheless, tilt testing can provided useful information on these patients and can be used to select patients responsive to cardiac pacing.

## Electrophysiological studies

The diagnostic yield of electrophysiological testing is highly dependent on patient selection. In patients with structural heart disease the yield is more than 50% whereas in patients without structural heart disease and a normal EKG diagnostic yield may be around 10% [5]. In addition, in patients with an abnormal EKG this type of investigation can reveal ventricular arrhythmias or bradycardia in 17% and 19% of patients respectively [2,18]. However, a negative test can not rule out an arrhythmic cause and has low predictive value, particularly in patients with non-ischemic cardiomyopathies, the long QT syndrome and the Brugada syndrome.

## Implantable loop recorders

Recently, implantable loop recorders have become available to study patients with unexplained recurrent syncopal events. This device has a solid state loop memory capable of storing electrocardiographic events up to 40 minutes before and 2 minutes after the activation. It can be easily placed subcutaneously under local anaesthesia and has a battery life of 15 to 18 months.

Krahn and co-workers [19] were among the first to describe a high diagnostic yield of the implantable loop recorder (ILR) in 16 patients with recurrent syncope. Extensive investigations including electrophysiologic studies, treadmill testing, 48-hours ambulatory monitoring and tilt table testing failed to obtain a definite diagnosis in these patients. In 94 % of cases recurrent syncope had occurred after implantation of the device revealing a arrhythmogenic cause in 60 %. Consequently in 40% of these patients no arrhythmias were detected. In all patients with an arrhythmogenic cause, successful therapy was implemented.

A final analysis was recently presented extending the follow-up to 40 ± 10 months [20]. From the 24 patients included, 52 % presented with an arrhythmic cause with bradycardia in the vast majority of patients. Treatment was directed at the underlying cause in the 18 patients who received specific diagnosis. During follow up, syncope did not occur in 16 of the18 treated. In 3 patients who underwent explantatition of the device after battery end-of-life without recurrent syncope, no recurrence was seen . The same group also presented a large series of 85 patients with recurrent undiagnosed syncope despite extensive evaluation [21]. Patients were eligible if they had at least to syncopal episodes within the previous 2 months or if they had a single syncope with a history of presyncope. In all ILR was implanted which resulted in detection of rhythms in 21 patients, the vast majority (18 pts) having a bradycardia. Of interest, in 29 patients no arrhythmia was detecteted despite symptoms. In addition, the inability to freeze the device occurred in 8 patients. Patients with syncope were more likely to record a arrhythmia during symptoms compared to patients with a history of presyncope (70 % vs 24 % p = 0.005).

A rather high proportion of patients (32 %) had no symptoms despite prolonged monitoring. This spontaneous resolution was also reported by others [20] who noted a 57 % - 80 % resolution of symptoms. Van Nierop et al [22] has recently reported on 35 patients who underwent implantation of an ILR because of recurrent syncope or presyncopal events. In 83 % of patients a symptom-rhythm correlation could be studied, revealing a clear arrhythmia in 23 %, half of them having bradycardia. Resolution of symptoms was presented in 70 % whereas the mean annual syncopal event rate was significantly decreased after implantation of the loop recorder (4.7 ± 2.4 vs 1.3 ± 0.7, p < 0.01). These patients were significantly younger and most of them were women. In 11 % patients were not capable of activating the device to save the event. This relatively large proportion of patients not capable of a proper activation of the loop recorder has led to the introduction of a second generation loop recorders capable of storing events after detection of a (programmable) heart rate. Although this may seem advantageous it might also introduce confounding factors since rhythms may be detected which are completely without symptoms. The predicted value of these rhythms have not been studied and should be interpreted with great caution [23].

Interpretation of the stored arrhythmia may also be difficult and is highly depended of the quality registration of QRS amplitude and P-wave morphology. Zellerhof and co-workers [24] compared the stored ECG in 4 different positions in 56 consecutive patients. The best ECG quality (defined as the highest QRS amplitude and the best visible P wave, QRS duration and QT interval) was found in the left or right sternal position with a horizontal position of the ILR.

## Limitations of the device

With increasing healthcare costs, a proper selection of patients for implantation of an implantable loop recorder is mandatory. Total costs of the investigation of patients with recurrent syncope is high [6] because of extensive diagnostic testing. Krahn et al [25] demonstrated that ILR implantation could reduce costs in a pilot study of 24 patients referred for recurrent syncope.

Although symptom-rhythm correlation can be obtained in the vast majority of patients, about 25 % of all patients remain without definite diagnosis despite the use of ILR. In addition a relatively large proportion of patients failed to freeze the device ranging from 11 % to 36 % of all studies which underscores the need for proper patient selection [21,22]. Previous studies demonstrated that patient selection should focus on those subsets with frank syncope which have a significantly higher diagnostic yield as compared to patients presenting with pre-syncope.

With the increasing use of the device other negative aspects have been demonstrated. De Cock and co-workers [26] investigated the potential interference from electronic article surveillance devices and found that interference may occur causing malfunction of this device. Cellular phones did not produce any interference. Finally the device is not able to monitor blood pressure, which can play a key role in the assessment of vasovagal syncope. It has been hypothesized that in the near future these devices will be capable of recording, apart from the arrhythmia, other physiological parameters (such as blood gas analysis) which may further increase the diagnostic yield [27,28].

In conclusion, the implantable loop recorder has been demonstrated to be a very useful tool in the work-up of patients with unexplained recurrent syncope "that is here to stay". Undoubtedly future development of the device will help us in the treatment of these patients.

## References

[R1] Heaven DJ, Sutton R (2000). Syncope. Crit Care Med.

[R2] Kapoor WN, Karpf M, Wieand S (1983). A prospective evaluation and follow-up of patients with syncope. N Engl J Med.

[R3] Day SC, Cook EF, Funkenstein H (1992). Evaluation and outcome of emergency room visits with transient loss of consciousness. Am J Med.

[R4] Linzer M, Yang EH, Estes M (1997). Diagnosing syncope: Part 2; Unexplained syncope. Ann Intern Med.

[R5] Linzer M, Yang EH, Estes M (1997). Diagnosing syncope: Part 1 Value of history, physical examination, and electrocardiography. Ann Intern Med.

[R6] Calkins GK, Byrne M, El-Atassi R (1993). The economic burden of unrecognized vasodepressor syncope. Am J Med.

[R7] Brugada J, Brugada R, Brugada P (1998). Right bundle branch block and ST segment elevation in leads V1 through V3: a marker for sudden death in patients without demonstrable structural heart disease. Circulation.

[R8] Camm AJ, Janse  MJ, Roden DM (2000). Congenital and acquired long QT syndrome. Eur Heart J.

[R9] Corrado Domenico, Basso Cristina, Thiene Gaetano (2000). Arrhythmogenic right ventricular cardiomyopathy: diagnosis, prognosis, and treatment. Heart.

[R10] Recchia D, Barzilai B (1995). Echocardiography in the evaluation of patients with syncope. J Gen Intern Med.

[R11] Benditt DG, Ferguson DW, Grubb BP (1996). Tilt table testing for assessing syncope. American College of Cardiology. J Am Coll Cardiol.

[R12] Kosinski D, Grubb BP, Karas BP (2000). Exercise-induced neurocardiogenic syncope: clinical data, pathophysiological aspects, and potential role of tilt table testing. Europace.

[R13] Parry SW, Kenny RA (2001). The role of tilt table testing in neurocardiocascular instability in older adults. Eur Heart J.

[R14] Kenny RA, Ingram A, Bayliss J (1986). Head-up tilt: A useful test for investigating unexplained syncope. Lancet.

[R15]  Benditt DG, Ferguson DW, Grubb BP (1996). Tilt table testing for assessing syncope: American College of Cardiology. Eur Heart J.

[R16] Connolly SJ, Sheldon R, Roberts RS (1999). The North American Vasovagal Pacemaker Study (VPS). A randomized trial of permanent cardiac pacing for the prevention of vasovagal syncope. J Am Coll Cardiol.

[R17] Benditt DG (1999). Cardiac pacing for prevention of vasovagal syncope. J Am Coll Cardiol.

[R18] DiMarco JP (1997). Value and limitations of electrophysical testing for syncope. Cardiol Clin.

[R19] Krahn AD, Klein GJ, Norris C (1995). The etiology of syncope in patients with negative tilt table and electrophysiological testing. Circulation.

[R20] Krahn AD, Klein GJ, Yee R (1998). Final results from a pilot study with an implantable loop recorder to determine the etiology of syncope in patients with negative noninvasive and invasive testing. Am J Cardiol.

[R21] Krahn AD, Klein GJ, Yee R (1999). Use of an extended monitoring strategy in patients with problematic syncope. Reveal Investigators. Circulation.

[R22] Nierop PR, van Mechelen R, van Elsacker A (2000). Heart rhythm during syncope and presyncope: results of implantable loop recorders. Pacing Clin Electrophysiol.

[R23] Waldo AL, Biblo LA, Carlson MD (1992). Ventricular arrhythmias in perspective: a current view. Am Heart J.

[R24] Zellerhoff C, Himmrich E, Nebeling D (2000). How can we identify the best implantation site for an ECG event recorder?. Pacing Clin Electrophysiol.

[R25] Krahn AD, Klein GJ, Yee R (1999). The high cost of syncope: cost implications of a new insertable loop recorder in the investigation of recurrent syncope. Am Heart J.

[R26] de Cock CC, Spruijt HJ, van Campen LM (2000). Electromagnetic interference of an implantable loop recorder by commonly encountered electronic devices. Pacing Clin Electrophysiol.

[R27] Brignole M, Menozzi C, Moya A (2001). International Study on Syncope of Uncertain Etiology (ISSUE) Investigators. Implantable loop recorder: towards a gold standard for the diagnosis of syncope?. Heart.

[R28] Kenny RA, Krahn AD (2001). Implantable loop recorder: towards a gold standard for the diagnosis of syncope?. Heart.

